# Co-design of an Augmented Reality Asthma Inhaler Educational Intervention for Children: Development and Usability Study

**DOI:** 10.2196/40219

**Published:** 2023-07-25

**Authors:** Antonia O'Connor, Andrew Tai, Malcolm Brinn, Amy Nguyen Thuc Hien Hoang, Daniele Cataldi, Kristin Carson-Chahhoud

**Affiliations:** 1 Respiratory and Sleep Department Women's and Children's Hospital Adelaide Australia; 2 School of Medicine The University of Adelaide Adelaide Australia; 3 Robinson Research Institute The University of Adelaide Adelaide Australia; 4 Translational Medicine and Technology Research Group University of South Australia Adelaide Australia; 5 Australian Centre for Precision Health South Australian Health and Medical Research Institute Adelaide Australia; 6 Paediatric Medicine The Women's and Children's Hospital Adelaide Australia

**Keywords:** asthma, asthma education, pediatric, pediatric asthma, co-design, usability, development, smartphone, tablet, augmented reality, health education, mobile app, mobile phone

## Abstract

**Background:**

Smartphone and tablet apps that deliver health care education have been identified as effective in improving patient knowledge and treatment adherence in asthma populations. Despite asthma being the most common chronic disease in pediatrics, there are few apps that are targeted specifically for children. Only half of children with asthma have acceptable control of their symptoms, and 40%-98% do not use their inhalers correctly. With children being increasingly connected to technology, there is an opportunity to improve asthma inhaler technique education by delivery via smartphone or tablet apps. Augmented reality (AR) technology was used in this study to capitalize on growing technological innovations. Digital health interventions that use a co-design process for development have the highest likelihood of successful uptake and effectiveness on their intended outcomes. Perceived usability also has been shown to improve the effectiveness of education as well as the acceptance of the intervention.

**Objective:**

The aims of this study were to describe the co-design process, development, and design outcomes of a smartphone or tablet app that incorporates AR technology to deliver asthma inhaler technique education to children with asthma. This study also aimed to provide a usability evaluation, using the System Usability Scale to inform our work and future research, and recommendations for others performing similar work.

**Methods:**

The development of the AR asthma inhaler technique education app was based on an iterative co-design process with likely end users (children with asthma, their caregivers, and health care professionals). This involved multiple stages: recruitment of end users for qualitative interviews and usability testing with a previously designed educational intervention, which used an AR-embedded smartphone or tablet app; ideation of content for a specific asthma inhaler technique education intervention with end users; development of the specific asthma inhaler intervention; and 2 further rounds of interviews and usability testing with the redesign of the initial prototype.

**Results:**

We included 16 participants aged 9-45 years. Using the co-design process, the AR asthma inhaler technique education app was designed, incorporating the preferences of end users. After iteration 1, animation was included based on the feedback provided. Iteration 2 feedback resulted in increased AR experiences and the removal of the requirement of a paper-based resource to trigger AR in the third iteration. Throughout all rounds, the ease of use of the app and the novel nature of the intervention were frequently described. The usability of the intervention overall was perceived to be excellent, and the mean System Usability Scale score of the intervention was found to be highest in the final round of evaluation (90.14).

**Conclusions:**

The results from this co-design process and usability evaluation will be used to develop a final AR asthma inhaler technique educational intervention, which will be evaluated in the clinical setting.

**International Registered Report Identifier (IRRID):**

RR2-10.1177/16094069211042229

## Introduction

### Background

There are currently over 6 billion smartphone users worldwide, with a 50% increase in the number of users over the last 5 years [1]. With the abundance of smartphone users and over 50,000 health care or medical apps available, the ease of access and convenience of such apps became clear during the SARS-CoV-2 pandemic, during which surveys found that 40% of respondents trialed new health care apps for monitoring their health [2,3]. Systematic reviews of health care education delivered via smartphone or tablet apps have identified effectiveness for outcomes such as improved knowledge, adherence to medications or treatment, and improved clinical care [4]. For asthma self-management, health care apps have also shown positive effects, with improvements in quality of life and asthma control [5,6].

Despite asthma being more prevalent in the pediatric population and only 50% of this population having acceptable control of their asthma symptoms, only 5% of the almost 150 apps available related to asthma are targeted specifically toward children [7-9]. This subgroup of patients with asthma is increasingly connected to digital technology, with the age of introduction continually dropping and research suggesting that some children are more familiar with devices, such as smartphone and tablets, than with books [10]. It is clear that smartphone and tablet-related apps should be designed for this population.

Smartphone and tablet apps that use augmented reality (AR) technology may provide a novel, generation-appropriate delivery mechanism for asthma self-management education in children. Using smartphone and tablets as viewing devices, AR technology has the ability to superimpose digital information over real-world objects, giving the impression of coexistence within the same space [11]. AR is one of the leading novel technological innovations in the medical and health care industry, with their adoption into medical education and training, diagnostic imaging, and patient management already being prominent [12-14]. While research of AR in asthma education is scarce, it has been shown in other sectors to have proven efficacy for behavior change [11,15]. AR has yet to be explored in an asthma pediatric cohort, with only 1 study to date reporting on the use of AR in inhaler education for children without asthma [16].

Although still described as a relatively new process [17], the co-design of digital health interventions facilitates active collaboration between intended end users, key stakeholders, and software developers to build a program with the highest likelihood of successful uptake and effectiveness on intended outcomes [18,19]. There is growing awareness about the importance of consumer co-design for tech-based health interventions among youth and the need to publish specific details of the consumer engagement process, enabling reproducibility and scientific rigor [18-22]. The risk of inadequate engagement is inferior and less appealing products and can lead to low uptake and effectiveness [22]. In 2016, Schneider et al [23] highlighted the importance of developing an app in collaboration with a cohort of young people with asthma. However, to our knowledge, few studies since then have been published on a user-centered design process in asthma [23-27].

In addition to co-design with potential end users and stakeholders, the usability of an intervention should also be evaluated through the design process. The International Organization of Standardization defines usability as “the extent to which the intervention or product can achieve specified goals by specified users with effectiveness, efficiency and satisfaction” [28]. Perceived good usability has been shown to improve the effectiveness of the education from the intervention as well as improve productivity and end user well-being in health-related apps [29,30]. Good usability perceived by clinicians is also vital for increasing the likelihood of successful uptake of the intervention within clinical settings, with poor perceived usability of technology-based systems, such as electronic health systems, linked to increased workload and lower acceptance of the system [31-34].

With the asthma inhaler technique having been well studied to be frequently performed incorrectly, with errors in 40%-92% of children, there is a clear ongoing need for alternative methods to deliver asthma inhaler technique education to this cohort [35-37]. Given the popularity of smartphones among young people and their increasing use, a smartphone or tablet app that uses AR may be an effective way to address this. With the small number of apps available for young people with asthma but the growing popularity of their usage, there is a need to co-design a smartphone or tablet app for this cohort. To maximize effectiveness, a co-design process that focuses on usability is necessary.

### Objectives

Our main objective was to undertake a co-design process for a smartphone or tablet app that uses AR technology to deliver asthma inhaler technique education, to capitalize on growing technological innovations in children with asthma and address the paucity of co-designed apps. Our secondary objective was to evaluate the usability of the AR smartphone or tablet app. As AR is a novel technology for delivering asthma education, the co-design process of an intervention and evaluation of its usability are necessary. Our aims were to describe the process, development, and design outcomes and perform usability evaluation to inform our work and future research, as well as to provide recommendations for others performing similar work.

## Methods

### Overview

We created an AR-enabled smartphone or tablet app to address the 40%-92% of children who have incorrect asthma inhaler technique. Development of the AR asthma inhaler technique education intervention was based on an iterative co-design process [38]. This involved 3 rounds of semistructured one-on-one interviews with likely end users to ideate content for the asthma inhaler technique education intervention initially and then obtain feedback to inform subsequent iteration development. Evaluation of the usability was performed in each round, using the System Usability Scale (SUS) questionnaire [39].

Qualitative evaluation based on the Theoretical Framework of Acceptability and Theoretical Domains Framework was obtained through interviews and questionnaires, with these results presented in separate papers [40,41]. In brief, the Theoretical Framework of Acceptability is a validated framework for the assessment of the acceptability of health care interventions to aid the identification of any characteristics that may be improved [41,42]. The Theoretical Domains Framework is also a validated framework that is used for the investigation of the barriers and facilitators of health behavior change interventions [43]. This paper will focus only on the iterative co-design process and usability, which are important to publish to enable reproducibility and scientific rigor.

### Ethics Approval

Ethics and governance were approved on August 21, 2020, after the study was reviewed by the Human Research Ethics Committee of the study site (approval number HREC/20/WCHN/74). Participants were provided with participant information sheets prior to enrollment, and written informed consent or oral assent was obtained from participants prior to interviews being conducted.

### Recruitment and Participants

An approximate total sample size of 15-20 participants was determined, prior to recruitment, for 3-4 usability testing rounds. This sample size was based on previous usability studies and experts of usability testing advocating that 5 users be involved per round, as 80% of usability problems can be found within these 5 users [44]. Five users per round was not a strict rule; however, as Faulkner [45] suggested, increasing the numbers tested can improve data confidence.

Purposive sampling was planned for recruitment to ensure adequate diversity of likely end users for maximal transferability of the intervention. Likely end users were children with asthma; their caregivers, who would likely be involved in the supervision and delivery of the intervention; and health care professionals (HCPs), who had experience in the management of children with asthma and would be those who introduced the intervention to children and their families. HCPs were also thought to be imperative in the co-design process and usability testing, as they would allow for knowledge and feedback on how the intervention would be able to complement standard care and be successfully integrated within the relevant settings. Purposive sampling was also used to ensure that a broad range of experiences, backgrounds, and opinions could be obtained from participants.

Inclusion criteria for children were an age of 8-17 years and a clinical diagnosis of asthma among those who were able to give assent. Inclusion criteria for caregivers were those who were the primary caregivers for children with a clinical diagnosis of asthma and were able to give consent. With purposive sampling intended to gain good representation of end users, it was identified midrecruitment that predominantly younger children were presenting to the hospital during the SARS-CoV-2 pandemic when recruitment was occurring, and there were minimal face-to-face outpatient clinic appointments also affecting recruitment from the older cohort. The decision was made halfway through recruitment to change the inclusion criteria to children aged 8-12 years, in consultation with experts in clinical care and technological innovation design.

Inclusion criteria for HCPs were nursing professionals, pediatric general medical doctors, respiratory doctors, pharmacists, asthma educators, or general practitioners who had treated and managed children with asthma regularly for over 12 months in the previous 5 years.

Potential participants who were non-English speaking were excluded. Participants were recruited by the primary investigator, starting from July 2021, at a tertiary pediatric hospital within Australia. Potential participants were approached and screened for inclusion and provided with patient information sheets. Potential participants were given time to review the information sheets and given the opportunity to decline participation.

### Data Collection and Analysis

Co-design data were obtained through one-on-one interviews, using semistructured moderator guides, by the primary investigator who had received interview training. Focus groups had been initially planned for the co-design process; however, due to the SARS-CoV-2 pandemic, this changed to one-on-one interviews. Interviews took approximately 20-40 minutes per participant and involved four components: (1) exploring the participants’ previous experiences with asthma and asthma education (specifically inhaler technique education), experiences with smartphone and tablet apps in health care, and experiences with AR; (2) being shown the intervention by the interviewer; (3) being able to use the intervention as a one-off; and (4) exploring participants’ views and experiences of the trialed intervention.

All interviews were audio-recorded, auto-transcribed, and check-backed by the primary investigator to ensure all data were verbatim. Feedback from each iteration was consolidated by the primary investigator and discussed with the project team, which resulted in an agreed set of changes over subsequent iterations. The evidence underpinning these recommendations has been presented in the results with supporting evidence from the quotes. We prioritized changes where there was some consensus by participants that a change was needed and changes that were within the scope of our budget and time.

Usability data were collected via the SUS questionnaire. The SUS is a common, simple, standardized questionnaire for perceived usability, which has been used since the 1980s [39,46]. It was chosen for this study due to its known suitability in the evaluation of computer systems, medical systems, and mobile devices; its relatively simple ease of administration; its ease of interpretation with known reference standards; and its suitability with small sample sizes [47]. Participants were asked to complete the questionnaire once the interview had been completed.

The standard approach for scoring the SUS was used, in which the 10 questions were answered based on the 5-point scale, odd-numbered items had 1 subtracted from the raw score, and even-numbered items had the raw score subtracted from 5, with the sum of the adjusted scores multiplied by 5 for the standard SUS score [39]. If a participant did not score an item, it was given a raw score of 3 [39]. The standard SUS scores were entered into Microsoft Excel to determine the mean, median, and SD for all participants. The higher the score, the better the usability, with Bangor et al [48] suggesting a system needs to score above 70 to be considered at least passable, and better systems will score in the high 70s to high 80s, with scores over 90 indicating a truly superior system. The SUS, which was supplied for children, had small wording modifications (Table 1).

**Table 1:** SUS^a^ for children.

Item	SUS statement	Modified statement for children
1	I think that I would like to use this system frequently.	I think that I would like to use this resource often.
2	I found the system unnecessarily complex.	I found the resource unnecessarily complicated.
3	I thought the system was easy to use.	I thought the resource was easy to use.
4	I think that I would need the support of a technical person to use this system.	I think that I would need the support of a technical person to be able to use this resource.
5	I found the various functions in this system were well integrated.	I found the various functions in this resource were put together well.
6	I thought there was too much inconsistency in this system.	I thought there were too many differences in this resource.
7	I would imagine that most people would learn to use this system very quickly.	I imagine that most people would learn to use this resource very quickly.
8	I found the system very cumbersome to use.	I found the resource very difficult to use.
9	I felt very confident using the system.	I feel very confident using the resource.
10	I needed to learn a lot of things before I could get going with this system.	I needed to learn a lot of things before I could get going with this resource.

^a^SUS: System Usability Scale.

## Results

### Participant Characteristics

A total of 16 participants were recruited between July 2021 and April 2022. This included 5 children with asthma, their 5 caregivers, and 6 HCPs who had experience in managing patients with asthma. There were 3 rounds, with 4 to 6 new participants per round (Table 2).

HCPs (n=6) included respiratory and general pediatrics doctors and nursing staff who had backgrounds of working within inpatient settings, emergency departments, and intensive care units, as well as educator roles within the hospital. Children (n=5) and their caregivers (n=5) with asthma had been predominantly diagnosed by a general pediatrician or respiratory specialist and were recruited while admitted into the hospital for the treatment of asthma exacerbations. All caregivers were female, and there was a broad range of educational levels, from not having completed year 12 to the completion of tertiary education.

**Table 2:** Number of participants per round of interviews.

	First iteration (N=4)	Second iteration (N=6)	Third iteration (N=6)
Health care professionals, n	2	2	2
Children with asthma, n	1	2	2
Caregivers of children with asthma, n	1	2	2
Sex (males:females)	0:4	1:5	4:2

### Co-design Process and Intervention Feedback

#### Iteration 1: Interviews and Feedback

**Figure 1:**
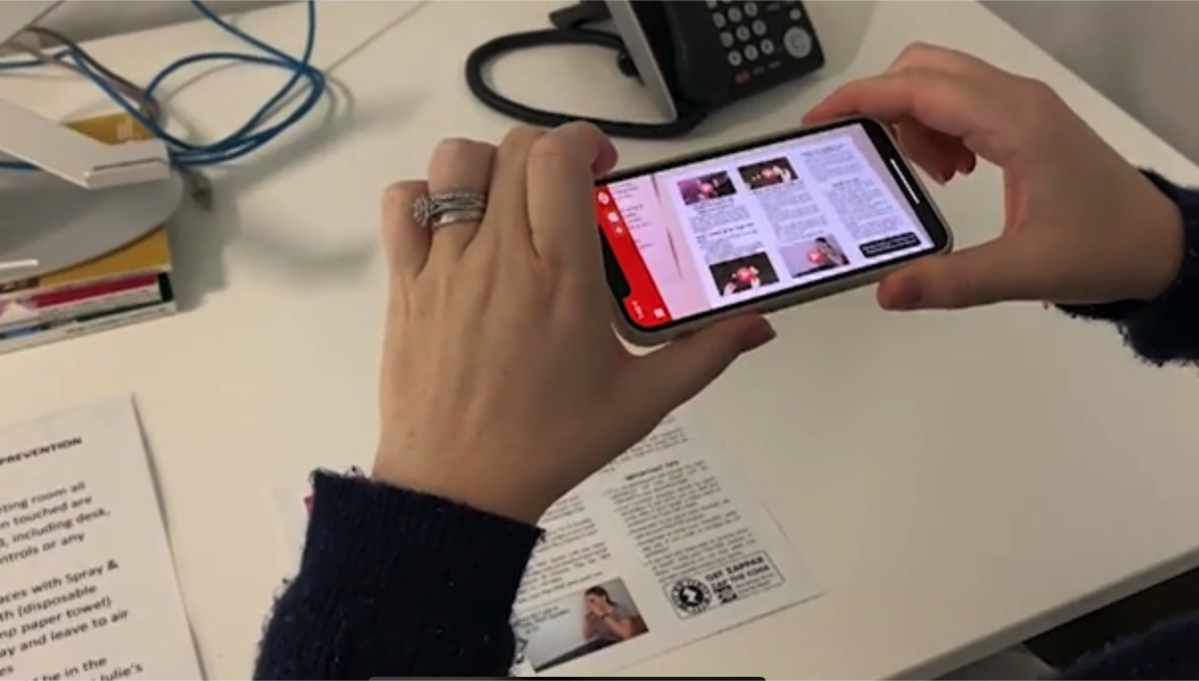
Iteration 1 demonstrated and used by participants in the first round.

Participants were initially shown an educational intervention, which incorporated AR to deliver education via a smartphone or tablet app on physiotherapy in cystic fibrosis. This was undertaken to provide a basic demonstration as to how AR-enabled technology delivered via a smartphone or tablet app works. The first round was aimed at the general concept of AR, as end-user input for a specific asthma inhaler educational intervention was wanted from the outset, with no previous published qualitative research, usability testing, or design processes for AR interventions in asthma education for children to form a precedence. The activation of digital content on physiotherapy equipment education was triggered (via pattern recognition) when the app was open and the smartphone or tablet hovered over a paper pamphlet. This allowed the user to have a direct view of video demonstrations on their smartphone or tablet device, giving the impression of the images on the paper “coming to life” (Figure 1). Participants were then given time to use the smartphone or tablet to trial the use of the intervention themselves, provide feedback on their experiences with the AR technology, and generate ideas on content specifically for an asthma inhaler technique educational intervention that used AR.

During the first round of interviews (N=4), the use of AR technology was found to be novel, interesting, and easy to use:

It was, it was new. It was good

caregiver, female

It’s like really being in the future…Like I wouldn't have ever, if you look at this piece of paper, you wouldn't expect that to kind of come to life. So I think that will be like the surprise factor as well, make it interesting for them and yeah. Get their attention

health professional, female

[it was] pretty easy to use

child, female

I liked it [augmented reality]

child, female

All participants reported that the use of an AR intervention would be a useful mechanism for delivering education to children and teenagers, including the child participant who answered “yeah” to the question “Do you think it would be useful for…learning about your asthma?” [child, female].

Suggestions and ideas for content were recommended through the interviews for the provision of education on all asthma inhaler devices as well as education on the asthma disease process itself:

It would good to have one, I guess, I mean, for each of the puffers or each of the puffer types like a metered dose inhaler and a spacer, and then like your, um, elliptas and kind of going through all of them with how to use them, that would be good

health professional, female

So particularly, um, use of inhalers, um, reliever and preventer, and sort of, um, a video representation to children of how they should use their preventer or their reliever

health professional, female

I guess that would be good to have a broad overview of asthma and what asthma is

health professional, female

To create content that younger people were more likely to engage with, the addition of animation and the need to improve relatability to children were suggestions that were provided to ensure that the content was more age appropriate:

somehow you have to make it sound exciting rather than just so factual

health professional, female

So I don't know if younger ones potentially, um, like cartoony...

health professional, female

The use of the paper pamphlet to trigger the AR and the digital educational content itself was also raised as a potential burden:

I guess the paper based resource has limitations in terms of, if you need the paper to use the app and if patients lose the paper, then it makes, has some issues…

health professional, female

#### Iteration 2: Interviews and Feedback

**Figure 2:**
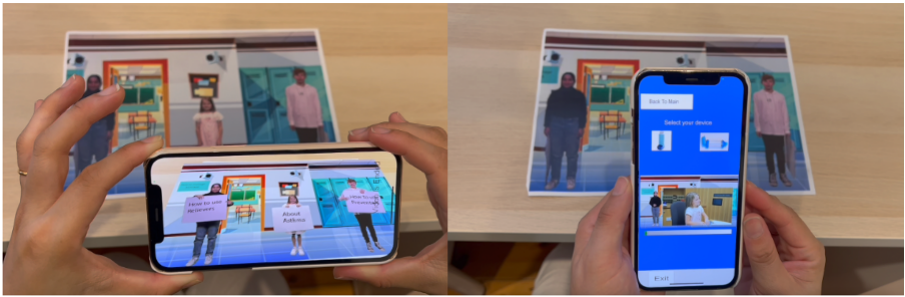
Iteration 2 demonstrated and used by participants in the second round.

**Figure 3:**
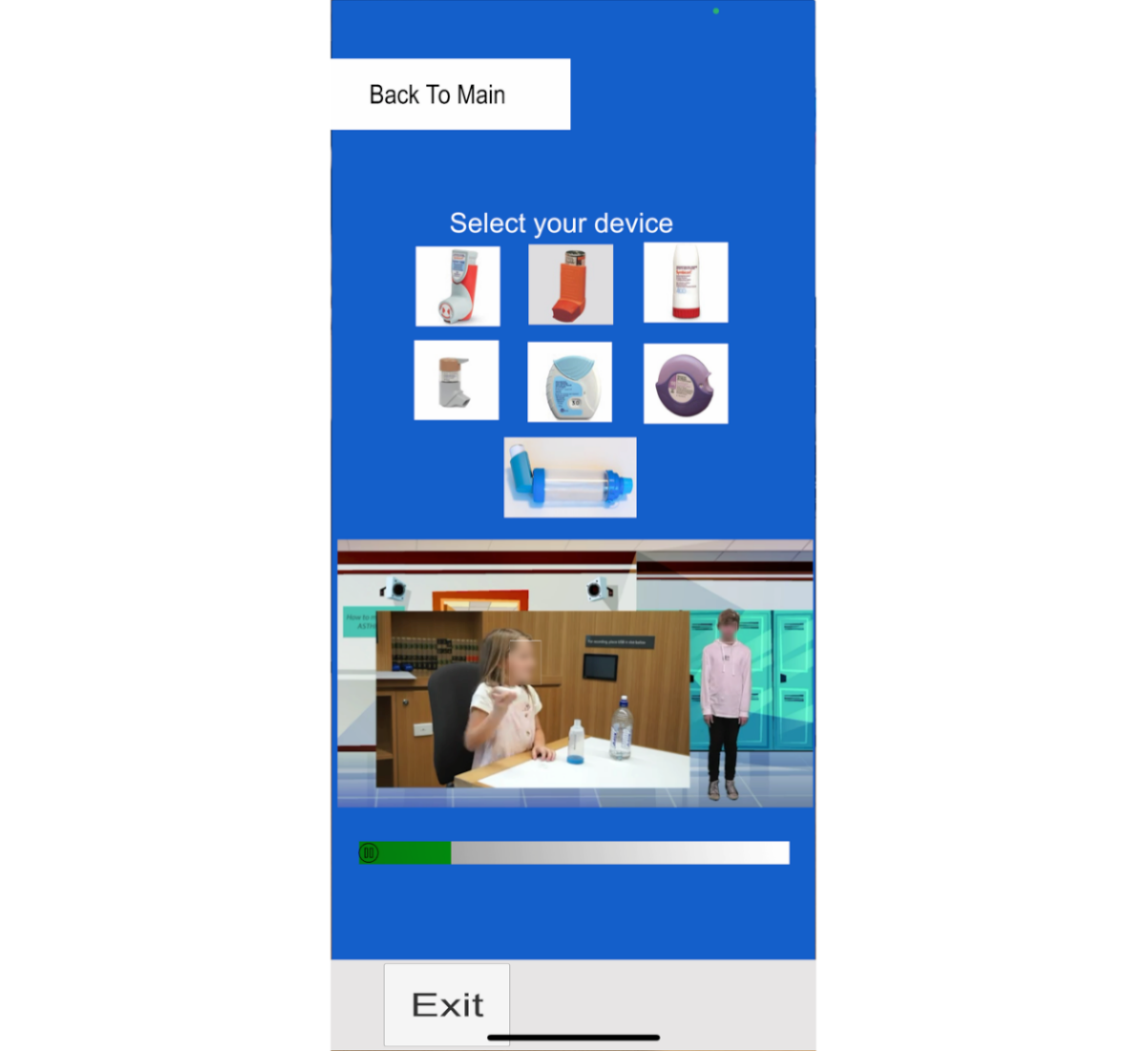
Iteration 2 demonstrated and used by participants in the second round - digital educational content for multiple asthma inhaler devices.

A second iteration, which was designed specifically for asthma inhaler technique education and used the same AR technology as iteration 1, was created (Figures 2 and 3). This iteration used the same paper-based mechanism to activate educational digital content, for which a poster of 3 children was created. When the smartphone or tablet app was open and hovered over the asthma-specific poster, the digital content—the 3 children “coming to life” and speaking on asthma and asthma inhalers—was triggered, with users being able to view this through the screen of their device, which superimposed the web-based educational content onto the real world. Users were given the option to click on signs being held by each child to view educational content on general asthma information, asthma reliever inhalers, or asthma preventer inhalers. If users chose to view content on asthma inhalers, further videos that demonstrated the steps on inhaler use were provided as options, with users being able to choose which inhalers they would like to learn more about (Figures 2 and 3). Due to time constraints, a purely app-based intervention was unable to be developed for iteration 2 (this was achieved by iteration 3), and hence, the same paper-based trigger mechanism was used. Components of feedback from participants that were addressed in this iteration included education on multiple inhaler devices and a broad overview of asthma, the use of animation, and the use of peer role models to improve relatability to young children. Multiple videos were created to demonstrate the use of the different types of inhalers, with users prompted to click on the inhaler that they wished to learn about. Scripts were written based on Lung Foundation inhaler technique videos, reviewed by asthma educators within the pediatric hospital, and had “readability” scores generated via Grammarly to ensure they were age-appropriate [49,50].

In the second round of interviews (N=6), participants received a demonstration of the AR asthma intervention and were then invited to use it themselves. After testing the intervention, feedback was again provided, and suggestions were made for improvements.

The technical functional aspects of the app were commented on, in regard to both the ease of use and the burden of the paper-based requirement for triggering and launching the app. The ease of the launch of the app was commented on by most participants (eg, “Easy to do, Just click” [caregiver, female]). However, technical issues related to functionality features of the paper-based resource were highlighted, which created difficulty in its use at times:

It would be, it would be good if once it started playing it, you didn't have to hold it there. Once you click out of the main menu

health professional, female

Maybe that once it's on, maybe it'll lock…because it's not comfy putting it over [the paper]

child, female

Asthma education delivered by children actors was received positively and thought to be a relatable means to deliver information:

my first thought is, oh, this is cool. It's actually kids doing it, which would really target kids. So I was like, oh, that's nice. It just makes it really relatable

health professional, female

as a kid with asthma, I think it makes it very relatable because you've got kids talking about it, kids demonstrating

health professional, female

Feedback and suggestions were also provided to improve engagement and decrease boredom for children. This was predominantly reflected in suggestions of animation incorporation, increased AR use, and gamification:

I do think that you have to [include] a game…just to teach them.

caregiver, female

It'd be good if it was more interactive because some people might have trouble listening to things.

child, female

Maybe you could like, have, um, maybe cartoon people next to them.

child, female

Just add a little bit more to the actual product…Come to life a little bit more then it grabs the kids

health professional, female

More components of asthma education were also requested in regard to content, such as expanding on asthma symptom triggers and the addition of asthma action plans:

One of the things that I think you should put on it is triggers

caregiver, female

you could go through steps of even like your asthma action plan

health professional, female

#### Iteration 3: Interviews and Feedback

**Figure 4:**
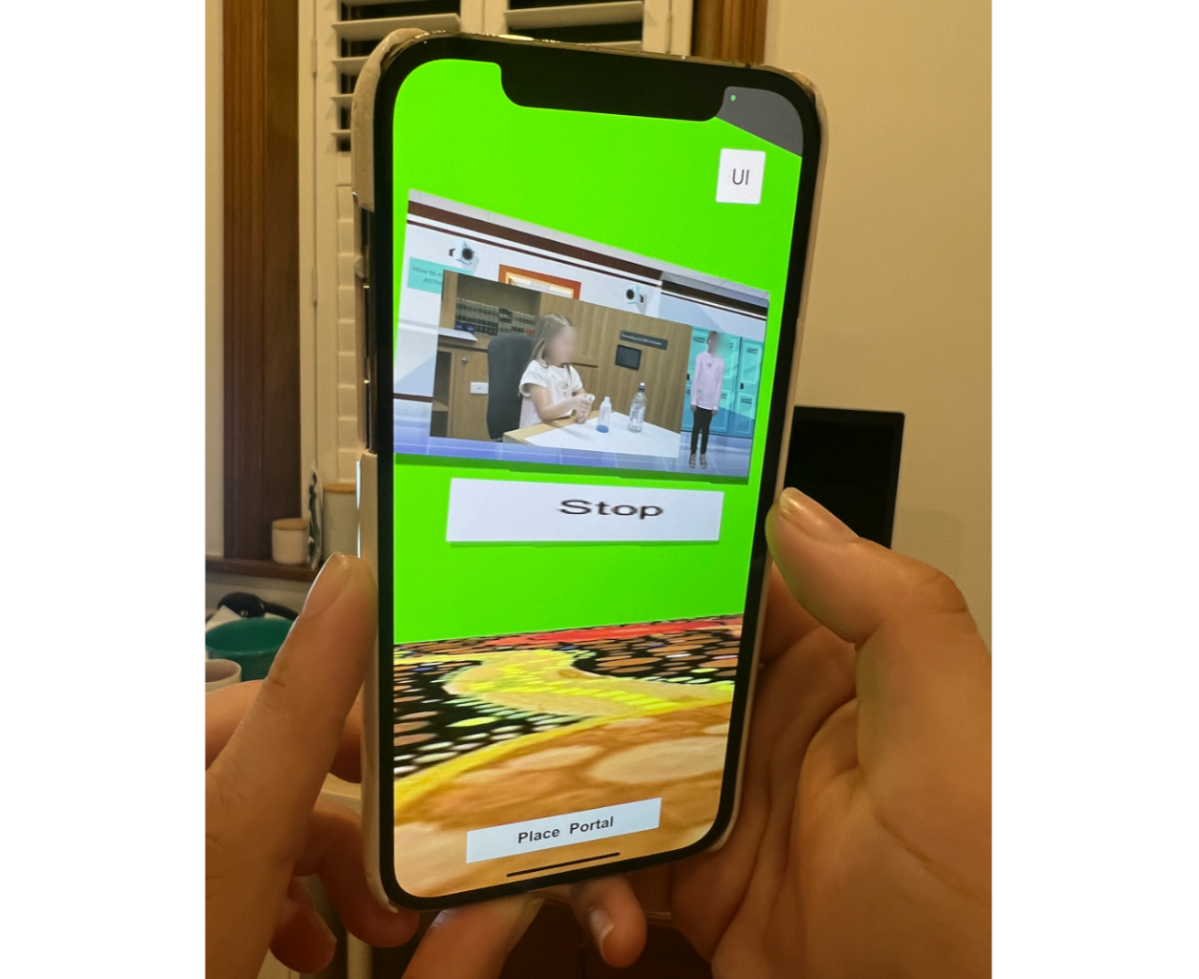
Iteration 3 demonstrated and used by participants in the third round—images after having entered a room through the portal and screens shown on the walls of the room with an asthma inhaler educational video.

**Figure 5:**
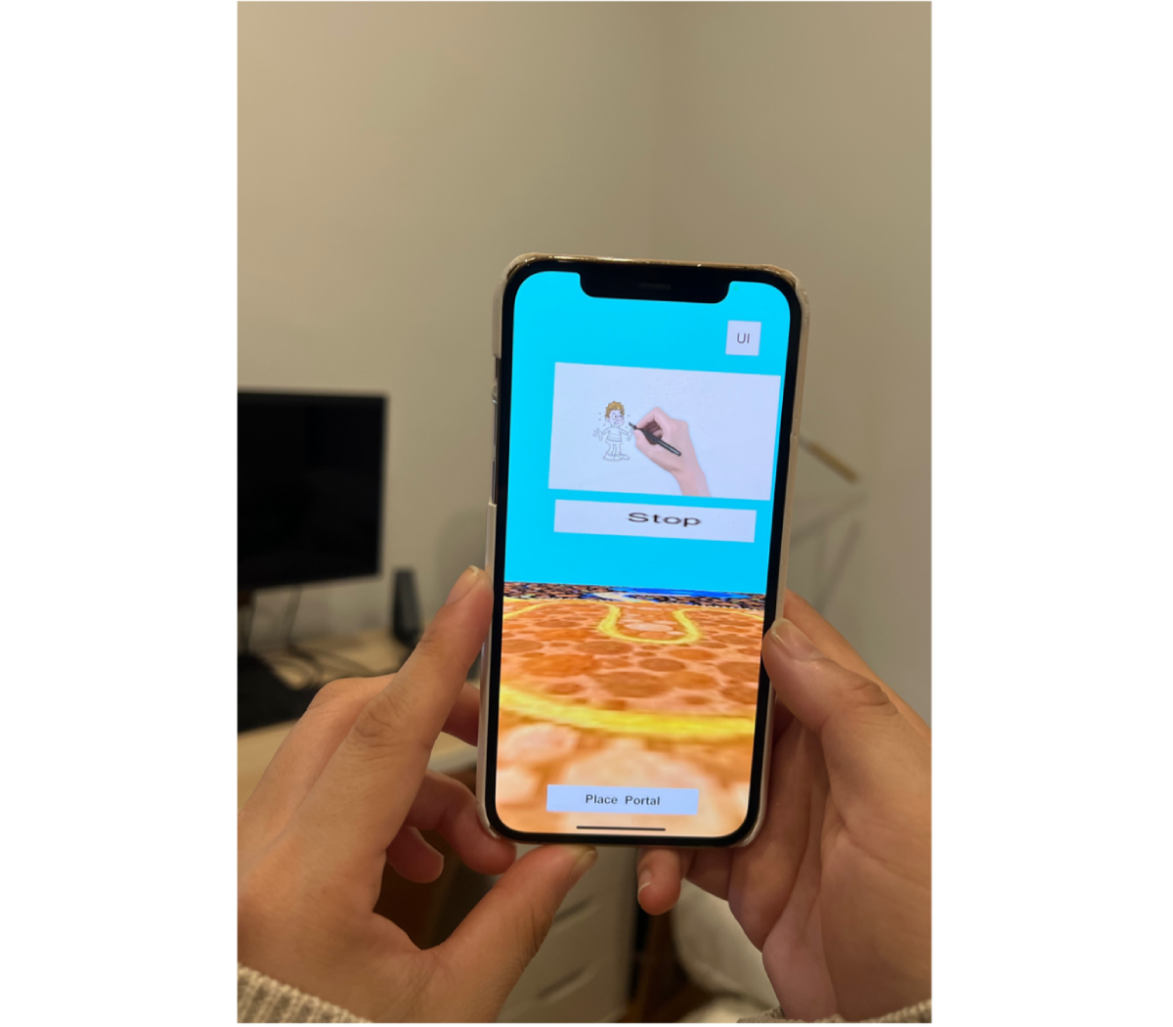
Iteration 3 demonstrated and used by participants in the third round—images after having entered a room through the portal and screens shown on the walls of the room with a whiteboard animation.

The third iteration of the AR asthma inhaler educational intervention was devoid of the paper-based trigger and modified, with additional major changes being the expansion of animation and the pivoting of the users’ AR experience through the smartphone to increase interaction of the app with young people. With the removal of the piece of paper required to trigger the smartphone or tablet app, an area of homogenous ground was used to trigger digital content instead. Once the app was opened, the user scanned any area of homogenous ground. This prompted the participant to place a “portal” onto the ground, which then allowed the participant to enter the “portal” and go into a room, which they could view on their smartphone or tablet. Asthma inhaler educational videos and animations were available on the walls of the room for participants to watch (Figures 4 and 5, Multimedia Appendix 1). This included educational content on general asthma information (eg, “What is asthma”), inhaled asthma reliever medications, inhaled asthma preventer medications, and the correct steps in the use of inhalers. Additional educational content on triggers was also added based on feedback; however, gamification was not yet incorporated into this iteration due to limitations on funding and the time it would take to create appropriate gamification for this particular intervention.

Six participants provided feedback in the third round of interviews after testing the AR smartphone or tablet intervention. An animated introduction using whiteboard animation was well received by all participants, as was the increased AR experience via the use of the portal and room:

The introduction was kind of fun. The playground and that stuff

child, male

the use of the drawings in the intro video, uh, was a great idea

health professional, male

You kind of just go into the portal and then the videos come up, I kind of like that you have to move the phone

caregiver, female

I liked the drawings

caregiver, female

Having children involved as the actors was once again highlighted as a positive to the intervention, as were the use of AR as a novelty technology and increasing the AR experience, which was suggested in iteration 2 to increase interactivity:

I think, I think the, uh, use of augmented reality is a novelty that kids would really connect with. Um, and the use of children delivering the education is also really good

health professional, male

I think it'll definitely add a component of something different and new to get them involved rather than just watching a video on a screen (regarding AR technology) [health professional, male];

it’s interesting (regarding AR technology)

child, male

I used to think health apps are like boring and that stuff but...reality is cool

child, male

Suggestions for improvement included incorporation of gamification again from children participants, as well as more animation within the intervention:

Animation? (when asked about how the intervention could be improved)

child, male

Little games and stuff? (when asked about how the intervention could be made more interesting or fun)

child, male

A lot of drawing animation is always, always good

caregiver, female

### Results of Usability (SUS)

All 16 participants who were recruited completed the usability questionnaire. Only 1 participant did not score all 10 items, and as described in the methods, these items were allocated a raw score of 3. SUS scores provided from participants ranged from 60 to 100, with an average of 87.65 (SD 16.96) and median of 88.75, indicating that the system was acceptable (scores of >70) and that the perceived usability of the intervention was excellent overall, with mean SUS scores between 85.5 (SD 12.17) and 90.4 (SD 11.7) considered within the “excellent” range when SUS scores have an adjective rating applied [51]. While in iteration 2, a child recorded an SUS score of 60; on review of raw data, this identified inconsistent scoring and responses (eg, scoring “agree” for both “I found the resource unnecessarily complicated” and “I thought the resource was easy to use”). This was also inconsistent with their interview transcript, with the participant reporting that the intervention was “easy to use,” suggesting that the scoring was likely incorrect.

When SUS scores were compared across the 3 rounds, the mean SUS score was lowest for iteration 2, and the highest mean SUS score was for iteration 3 (ie, the final round). Per Bangor et al [48], based on the SUS mean score, iteration 3 was classified as a truly superior system (Multimedia Appendix 1).

When SUS scores were compared across the 3 participant groups (children with asthma, their caregivers, and health professionals), health professionals scored the intervention highest in terms of perceived usability, with a mean score of 89.58 (SD 5.34), and caregivers scored it the lowest, with a mean score of 85.5 (SD 12.17) (Table 3). Of note, all scores were still within the “excellent” usability range across the 3 participant groups.

**Table 3:** SUS^a^ scores per participant group.

Participant group	SUS, mean (SD)	SUS, median (IQR)
Children with asthma	87.5 (16.96)	95 (60-100)
Caregivers of children with asthma	85.5 (12.17)	92.5 (70-97.5)
Health professionals	89.58 (5.34)	87.5 (85-100)

^a^SUS: System Usability Scale.

## Discussion

### Principal Results

This paper is the first to describe the co-design process of an AR asthma inhaler educational intervention for children. End users were engaged in the development from the beginning of the process, which allowed for a user-centered design. Participants had mostly favorable views of the AR intervention, with the ease of use of the technology and the novel nature of AR being able to capture the attention of children for inhaler technique education in all 3 iterations. Through the use of the iterative co-design process, the preferences of end users were also able to be incorporated with key suggestions, such as the addition of animation and increased interactivity with AR included to later iterations. With this process, it was possible to identify areas that required improvement or were perceived to not be necessary (such as the use of the paper-based resource to trigger the AR intervention) and provide information on the preferences of end users to inform further development of the intervention. The use of an iterative co-design process was particularly important for the development of this AR intervention in children for two main reasons: (1) the novel nature of AR as an educational delivery mechanism in health care education, especially for asthma in children, and (2) evidence showing that these design processes increase the efficacy and uptake of the intervention by end users [25].

Through use of the SUS, this intervention was found to have excellent perceived usability, with an overall mean of 87.6. The third iteration had the highest mean of 90.14, indicating a truly superior system, providing encouraging evidence that with the iterative co-design process, the intervention can continue to be improved on throughout subsequent rounds.

### Comparison With Prior Work

This paper aimed to describe a user-centered design and the usability of an AR intervention, which was delivered via a smartphone or tablet. To date, there have been no studies identified that describe a co-design process for asthma educational interventions that use AR or the usability of such developed interventions, as in this study.

Smartphone apps, which have poor usability and do not use this design process, have lower adoption rates, and despite the increasing use of mobile apps for health care education, only a small number of papers recently have described a co-design process or usability testing for asthma apps for children and young people [52]. Sonney et al [25] recently described, in 2022, using a “human-centered design” for refinement of an app, which was designed for asthma monitoring and as a behavioral intervention to promote shared asthma management between a parent and child with asthma. While children and their parents were involved in the process, their involvement from the outset in the design of the app was not apparent [53]. Mayoral et al [54] also recently described end-user involvement in the development of a mobile health app for children with asthma; however, children and adolescents were not involved until the later stages of its development. Other studies, such as one by Davis et al [24], described using a participatory approach from the preintervention development phase for an asthma self-management smartphone app; however, this was targeted at people aged 15-25 years with asthma, who would likely have differing preferences compared to our patient cohort. In regard to usability testing for asthma apps for children, Mayoral et al [54] also used the SUS for usability testing; however, Schneider et al [23] used semistructured interviews by a research assistant. While there is more recent literature on usability testing for asthma apps aimed at adults and adolescents with asthma, usability testing in children remains scarce [55-57].

### Limitations

Limitations for this study first lie with the limited generalizability of the intervention. Participants were only recruited if they were primarily English-speaking and were recruited from a tertiary pediatric hospital. Children and their caregivers were recruited during a hospital admission, indicating that the end users recruited were predominantly children who may have had more severe or more poorly controlled asthma and may have had a stronger desire for interventions to improve their asthma education. Similarly, health professionals were also recruited within the tertiary hospital setting, which may have led to the recruitment of health professionals who see and manage patients with asthma who have poorer control and are on the more severe end of the spectrum. In addition, 11 of 16 participants were female (69%); it is possible that feedback and app development would have been different with a more even distribution of genders. Although we had intended on purposive sampling, we were limited to the demographic of patients presenting to the site. During the recruitment phase, it was noted that children who were younger were presenting to the site and recruited. To ensure that purposive sampling was completely adhered to, a decision had to be made as to whether to try and increase the sampling size by adding a second site or adjusting the parameters of the age inclusion criteria. Following consultations with experts in clinical care and experts in technological innovation design, it was decided that we would have more targeted information if we focused on the younger cohort alone, and it was likely that the intervention would have had different requirements and feedback from older children (13-17 years inclusive). Another study for the older cohort is planned.

The small sample size of our study also limited the interpretation of the SUS; however, the usability evaluation of this study was for hypothesis-generating purposes. More research is required with a larger sample size to evaluate the intervention’s usability in the clinical setting. Another limitation in the evaluation of usability was that while the SUS can be used as an aid for understanding the overall level of the usability of an intervention, it does not necessarily identify detailed information on the intervention’s effectiveness or efficiency, which may be able to provide more information for improvement on subsequent iterations [58]. Lastly, while the wording of our SUS instrument was modified slightly for improved understanding among children, this had not been tested or validated previously, decreasing its reliability.

Not all feedback from interview rounds were able to be incorporated into the subsequent iterations, which is a further limitation to our study. Due to time constraints, we were not able to make adjustments to a paper-free version until iteration 3, so during iteration 2, we continued with the paper-based triggering model. There still might be advocation for a paper-based resource however, such as within hospital settings where pamphlets for education are predominantly used or for people who are reluctant to rely solely on technology-based resources. The incorporation of gamification was also not achieved during this study due to time and funding constraints; however, it should be strongly considered for smartphone or tablet asthma education app developers to increase engagement and interactivity with children with asthma. It is possible that had we been able to act on all identified feedback themes, SUS scores may have been higher.

Lastly, this study reports on only the design process and usability, but for the successful uptake and implementation of an intervention on a larger scale, other aspects of the intervention must also be evaluated, such as the acceptability of its use, the barriers and facilitators to its use, the feasibility of the intervention, and its efficacy. These will be addressed in future papers and studies.

### Conclusions

Not only is published research on the use of AR for asthma educational interventions in children scarce, the use of a co-design process in the development of smartphone or tablet asthma educational intervention apps, as well as the usability of such apps, is also infrequently reported. This contributes to the literature by identifying and incorporating the preferences of intended end users. The key recommendations found in our work, which should be considered by others in the design of similar interventions, are the inclusion of animation, increased interactivity, and gamification. This paper highlighted the importance of co-design and showed the improvement in usability scores with the incorporation of end user feedback through subsequent iterations of design. The results from this process are now being used to develop the final AR intervention for evaluation in the clinical setting.

## Appendix

Multimedia Appendix 1 Video demonstration of augmented reality intervention.

## Abbreviations

AR: augmented reality
HCP: health care professional
SUS: System Usability Scale
